# Estimation of microbial phosphate-accumulation abilities

**DOI:** 10.1038/s41598-018-37752-8

**Published:** 2019-03-19

**Authors:** Ajeeta Anand, Hideki Aoyagi

**Affiliations:** 1 Institute of Life Sciences and Bioengineering, Graduate School of Life and Environmental Sciences, University of Tsukuba, Tsukuba, Ibaraki 305-8572 Japan; 2 Faculty of Life and Environmental Sciences, University of Tsukuba, Tsukuba, Ibaraki 305-8572 Japan

## Abstract

Phosphate binders and dialysis can have harmful side-effects during the treatments of hyperphosphatemia. Therefore, we evaluated the capability of intestinal bacteria (lactic acid bacteria and bifidobacteria) as phosphate-accumulating organisms (PAOs) for phosphate accumulation, with the aim of determining whether PAO-formulated food can prevent hyperphosphatemia in the early stages. However, methods for estimating microbial phosphate-accumulation capacities require significant improvements regarding specificity, cost, and simplicity. The presented method analyzed cell-free broth to assess the phosphate accumulation capability of cells. Active cells and the constructed phosphate-deficient cells were incubated in assay salt media. After incubation, phosphate-deficient cell-free broth was taken as sample and the blank was the active cell-free broth. Therefore, effects of interfering agents and other metabolites were avoided and enhanced the specificity remarkably. Phosphate contents were assessed by reactions with toluidine blue O. In contrast to the case in previous studies, the shift in the first absorbance peak was found to be inversely proportional to the phosphate concentration. The minimum detectable phosphate concentrations for the 11th isolate of *Lactobacillus casei* JCM 1134 and 8th isolate of *Bifidobacterium adolescentis* JCM 1275 were determined to be 1.24 and 0.4 mg/L, respectively. Further, the validation results were found to be significant (p-value < 0.05).

## Introduction

In many developed countries, poor dietary habits and occupational stress have led to the wide prevalence of chronic kidney disease (CKD). One of the major effects of CKD is hyperphosphatemia, which occurs due to anomalies in kidney function. Hyperphosphatemia leads to several other diseases, including hypocalcaemia, renal bone disorders, and the abnormal calcification of the vasculature^1^. Current popular treatments for CKD-linked hyperphosphatemia include reducing the dietary intake of phosphorus, phosphate-binding chemotherapy, and the removal of phosphorus by dialysis. However, these affect the quality of life as they can result in heavy metal deposition, involve strict dialysis schedules, and can lead to hypercalcemia and other metabolic anomalies^2^.

Medical treatments prescribed during the later stages of CKD, when the kidney damage is irreversible, can lead to a serum phosphate imbalance and further impair the quality of life. However, if intestinal bacteria were found to exhibit good phosphate-removing properties, they could potentially be used for preventing hyperphosphatemia in the initial stages of CKD, as it is known that care is better than cure. Probiotics-based functional foods can be obtained without a prescription from a medical doctor and are consumed worldwide on a daily basis. In addition, phosphate-removing probiotics may offer a safer and healthier prevention strategy owing to their associated gut-health benefits, which also help maintain the quality of life.

With this in mind, the phosphate-removal properties of potential phosphate-accumulating organisms (PAOs) need to be evaluated. However, for determining the amount of polyphosphate (poly-P) stored in microbial species, the existing estimation methods are not suitable.

Phosphate extraction and quantification have been performed previously for estimating the total phosphate content within cells. The American Public Health Association proposed three standard digestion methods^3^ for determining the phosphate content. These include using perchloric acid, using nitric–sulfuric acid, and persulfate oxidation. The perchloric acid method is the most hazardous (strong acids such as HClO_4_ and HNO_3_ are used in this method) and time-consuming (many steps are involved) one and is preferred for samples containing solid matrices such as sediments or soil. The nitric–sulfuric acid method is recommended generally. However, with respect to wastewater research, the persulfate oxidation method^4^ is the simplest and most rapid approach (the treatment time is approximately 2 h)^5^.

During a standard procedure of phosphate extraction method, cell lysis is performed chemically. However, the hazardous chemicals involved in the process can cause undesirable changes and require the use of a costly disposal system. The impurities (such as proteins and nucleic acids) present in samples can affect the results of the extraction procedures. Further, the damage to poly-P chain length during the cell disruption procedure must be minimized. For example, Aravind *et al*. used NaOH and HCl to extract poly-P and this caused undesirable modifications^6^. To date, no extraction procedure that is free of undesirable side-effects has been reported.

Standard methods for the quantification of the orthophosphate content have also proposed by the American Public Health Association^3^. However, these methods have limited efficacy because of interfering agents. In the case of the vanadomolybdophosphoric acid and stannous chloride method, arsenates, thorium, bismuth, sulfides, thiosulfates, and molybdates act as interfering agents^3^. Further, the applicability of the ascorbic acid method is limited by the presence of antimony in potassium antimony tartrate, which is used as the reagent, as well as by the ionic strength of the sample, the acid concentration and the presence of persulfates^7^. Furthermore, while a high antimony concentration improves the method’s sensitivity^8^, it also causes a five-fold increase in the turbidity and decreases the reproducibility^7^.

The specificity of phosphate-estimation methods predominantly depends on the blank used, especially in the case of microbial species. Most methods use distilled water for the blanks. However, the poly-P content is estimated using the lysed cell-free extract (obtained using chemicals or solvents) after some degree of purification, so a relatively large amount of intracellular metabolites is still present. Therefore, distilled water is unsuitable for the blanks as far as the specificity is concerned. Furthermore, PAOs are microbial consortia that vary metabolically during the enrichment or cultivation stages. Therefore, during poly-P extraction, the metabolic dynamics of the enriched or cultivated PAOs cannot be determined or assessed against a blank of distilled water, as this may lead to false positive or negative results.

Recently, toluidine blue O (TBO) dye has been used for the spectroscopic poly-P estimations of extracts of cell-free algal hydrolysate using chloroform/isoamyl alcohol (2:1)^9^. Although this method has low sensitivity owing to the use of a lower reaction temperature (room temperature), its sensitivity can be improved merely by increasing the reaction temperature. On the other hand, the specificity and reliability of the method are limited if one uses distilled water for the blanks for the microbial species. Moreover, the use of a specific degree of phosphate polymerisation^9^ further restricts its applicability in the case of unknown poly-P samples.

The main aim of the present study was to evaluate the phosphate-accumulation capacities of PAOs that are neither affected by the medium used nor by the environmental conditions. To determine the least-affecting conditions for the assessment of the phosphate-accumulation capacities of microbes, we formulated a simple synthetic media which contained a few salts in order to maintain the osmolarity of the PAOs. In this study, the phosphate-accumulation capacities of probiotic bacteria were assessed. Such bacteria have great potential for use in preventive therapies for CKD patients who suffer from hyperphosphatemia. An indirect method wherein the supernatant was used instead of an extraction procedure and was employed to assess the phosphate-accumulation capacities of the probiotics (isolates of *Lactobacillus casei* JCM 1134 and *Bifidobacterium adolescentis* JCM 1275), in an attempt to overcome the challenges with the currently available microbial phosphate-estimation methods. Cells were transformed into phosphate-deficient cells to estimate the phosphate accumulation capability in a later assay. After the incubation in the latter assay, supernatants of phosphate-containing cells were used as blank samples to avoid interfering agents and to counterbalance other metabolic concentrations while supernatant of phosphate-deficient cells was considered as test sample. After phosphate accumulation, the remaining phosphate content was estimated via a reaction with TBO dye.

## Results

### Strategy for isolation of PAOs

L11 and B8 are the 11^th^ and 8^th^ isolates of *L. casei* JCM 1134 and *B. adolescentis* JCM 1275, respectively. These isolates were obtained through a three-step selection procedure (namely, phosphate enrichment, survival in low pH and separation through density gradient centrifugation in Percoll^®^, and a semiquantitative petri-plate method involving the formation of a TBO-phosphate complex in a culture colony), resulting in the desired phosphate-accumulating organisms. The components of the phosphate-enrichment medium were determined which were based on previous studies that aid phosphate-accumulation by microbes. That the PAOs were present in a high concentration in the phosphate-enrichment medium was confirmed through qualitative and quantitative methods. To remove the non-PAOs, the “survival in low pH” strategy was employed, wherein PAOs that can survive in low pH who can defend the low pH by releasing negatively charged phosphate ions in the medium and raising the pH by metabolising the stored polyphosphate as a phosphorous source. Next, the remaining non-PAOs were separated by density gradient centrifugation in Percoll. The suitability of this strategy was confirmed through qualitative and quantitative phosphate content assessment methods as well as cell survival measurements. In the third step, a petri-plate method was adopted wherein TBO dye was added to the synthetic medium with and without the phosphate, turning the grown colonies coloured. The selection of the elite PAOs was based on the size of the coloured zones that formed in the colonies.

### Preparation of phosphate-deficient cells

Active cultures were incubated in the transformation medium as well as in an ice box. The metabolic activities of the cells (in terms of the decrease in pH and the CFU/L and optical density (OD) values) along with their phosphate contents were monitored over 8 h of incubation, as shown in Tables 1 and 2. The metabolic activities of the cultures in the ice box nearly ceased. However, the active cell counts remained constant, as shown in Table 2, possibly because of the low temperature and dormancy. The cultures incubated in the ice box also maintained their sugar content (Table 2); this was probably because the metabolic activities had almost ceased and the broth contained enough sugar for the maintaining the needs of the cells. The cultures incubated in the transformation medium (without phosphate) maintained their active cell counts and were observed to be metabolically active, probably because the internal phosphates were used to maintain cellular metabolism (Table 2). Phosphate utilisation or phosphate-deficient cells were confirmed by the phosphate quantitative estimation method (Table 2). Thus, after 8 h of incubation, the transformation medium changed the cultures into phosphate-deficient cells (through the utilisation of the internal phosphate) while maintaining the sugar content and active cell count.

**Table 1:** Growth of cells incubated in transformation medium (that convert phosphate rich to phosphate deficient cells) were monitored over time where optical density (OD) and pH data are represented.

Probiotic	L11 of *L. casei* JCM 1134		B8 of *B. adolescentis* JCM 1275	
Time (h)	OD at 680 nm	pH	OD at 680 nm	pH
0	7.20 ± 0.020	6.01 ± 0.01	5.80 ± 0.002	6.02 ± 0.01
1	7.22 ± 0.010	6.05 ± 0.03	5.84 ± 0.005	6.01 ± 0.03
2	7.21 ± 0.002	6.03 ± 0.01	5.78 ± 0.012	5.94 ± 0.02
4	7.32 ± 0.012	5.98 ± 0.02	5.68 ± 0.002	5.87 ± 0.02
6	7.29 ± 0.004	5.91 ± 0.01	5.56 ± 0.008	5.82 ± 0.01
8	7.21 ± 0.010	5.86 ± 0.02	5.40 ± 0.010	5.77 ± 0.01

**Table 2:** Metabolic activities of cells incubated in transformation medium where CFU/L values and sugar and phosphate contents are represented.

Probiotic	L11 of *L. casei* JCM 1134			B8 of *B. adolescentis* JCM 1275		
Time (h)	CFU (10^11^) /L	Sugar content (μg/mg-dcw)	Phosphate content (mg/mg-dcw)	CFU (10^8^) /L	Sugar content (μg/mg-dcw)	Phosphate content (mg/mg-dcw)
0*	9.84 ± 0.02	36.5 ± 0.1	0.84 ± 0.03	8.92 ± 0.03	32.1 ± 0.1	0.73 ± 0.02
8*	9.39 ± 0.01	36.7 ± 0.0	0.33 ± 0.02	8.80 ± 0.02	32.4 ± 0.1	0.21 ± 0.01
0^#^	9.95 ± 0.01	36.4 ± 0.1	0.82 ± 0.02	8.95 ± 0.01	32.2 ± 0.0	0.75 ± 0.01
8^#^	9.87 ± 0.02	36.5 ± 0.1	0.79 ± 0.01	8.86 ± 0.02	32.2 ± 0.0	0.72 ± 0.01

Transformation medium and ice box are denoted by (*) and (#), respectively. Numerical values are expressed with standard deviations while “dcw” stands for dry cell weight.

### Incubation in salt solutions

**Figure 1:**
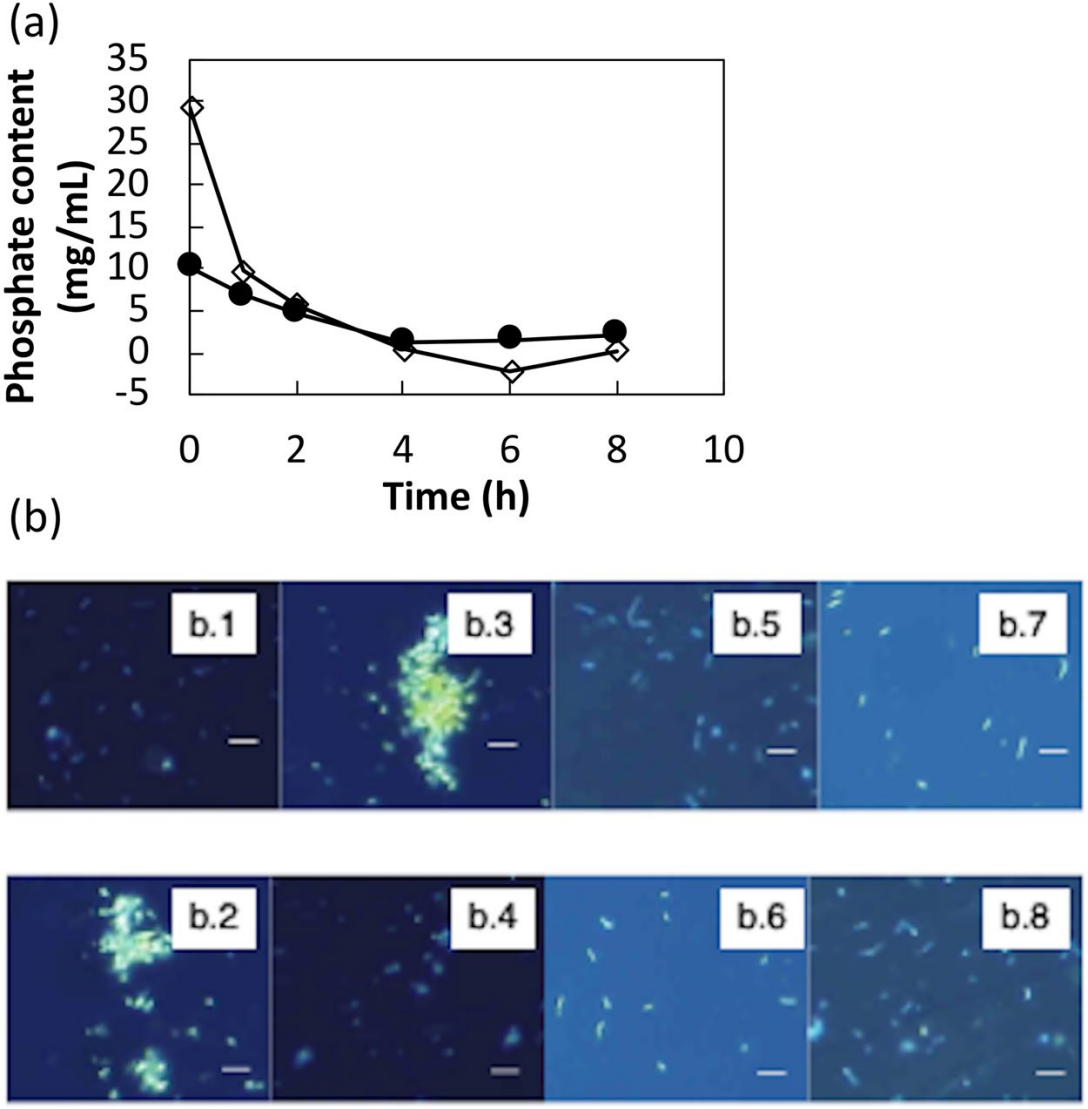
Metabolic activities of cells incubated in assay salt solution (which evaluated the phosphate accumulation abilities of cells) and monitored over time: (**a**) phosphate contents of supernatants of cells incubated in assay salt broth [(+P): ◊→L11 of *L. casei* JCM 1134 and •→B8 of *B. adolescentis* JCM 1275]. Standard errors are less than 8%. (**b**) Pre- and post-incubation DAPI micrographs of probiotics; b.1, b.2, b.5, and b.6 were incubated in phosphate-containing assay salt solution while b.3, b.4, b.7, and b.8 were incubated in phosphate-free assay salt solution. b.1→ at 0 h phosphate-deficient L11 of *L. casei*  JCM1134, b.2→ at 6 h L11 of *L. casei* JCM1134 (phosphate metabolising), b.3→ at 0 h active L11 of *L. casei* JCM1134, b.4→ at 6 h L11 of B8 of *B. adolescentis* JCM1275 (phosphate storing), b.5→ at 0 h phosphate-deficient B8 of *B. adolescentis* JCM1275, b.6→ at 6 h B8 of *B. adolescentis* JCM1275 (phosphate metabolising), b.7→ at 0 h active B8 of *B. adolescentis* JCM1275, b.8→ at 6 h B8 of *B. adolescentis* JCM 1275 (phosphate storing). White bars represent 2 μm.

NaCl was added to assay salt solution instead of a phosphate to create blank for the remaining phosphate-content measurements in the cell free assay salt solution owing to the fact that the absorbance of NaCl in the reaction product was negligibly low. Phosphate-deficient and phosphate-containing cultures were incubated in the salt solution with and without phosphate (K_2_HPO_4_) respectively, and their metabolic activities, OD and pH values, and phosphate contents were monitored over 8 h of incubation, as shown in Fig. 1a and Table 3. The phosphate contents were monitored qualitatively using DAPI staining to confirm phosphate accumulation (Fig. 1b). Incubation for 6 h and 4 h in the salt solution was found to be suitable for estimating the phosphate-accumulation capacities of the L11 isolate of *L. casei* JCM 1134 and the B8 isolate of *B. adolescentis* JCM 1275, respectively, in the salt solution (Fig. 1a), as significant cell losses were observed for periods longer than these Table 3).

**Table 3:** Metabolic activities of PAOs in assay salt solution and were monitored over time where optical density (OD) and pH data are represented.

Probiotic	L11 of *L. casei* JCM 1134				B8 of *B. adolescentis* JCM 1275			
Assay solution	+P		+NaCl		+P		+NaCl	
Time (h)	OD at 680 nm	pH	OD at 680 nm	pH	OD at 680 nm	pH	OD at 680 nm	pH
0	6.90 ± 0.020	6.02 ± 0.02	7.00 ± 0.003	6.01 ± 0.02	5.40 ± 0.013	6.03 ± 0.02	6.02 ± 0.002	6.01 ± 0.02
1	6.88 ± 0.010	6.04 ± 0.02	6.98 ± 0.020	6.02 ± 0.01	5.38 ± 0.004	6.03 ± 0.01	5.95 ± 0.001	6.02 ± 0.01
2	6.89 ± 0.012	6.01 ± 0.01	6.90 ± 0.014	6.00 ± 0.01	5.37 ± 0.006	6.00 ± 0.01	5.96 ± 0.012	5.98 ± 0.01
4	6.86 ± 0.006	5.99 ± 0.01	6.81 ± 0.016	5.98 ± 0.02	5.35 ± 0.004	5.97 ± 0.02	5.93 ± 0.011	5.94 ± 0.01
6	6.81 ± 0.002	6.17 ± 0.01	6.89 ± 0.009	6.10 ± 0.03	5.23 ± 0.005	5.89 ± 0.03	5.98 ± 0.013	6.03 ± 0.03
8	5.60 ± 0.006	6.23 ± 0.03	5.83 ± 0.004	6.18 ± 0.01	4.89 ± 0.002	5.82 ± 0.01	6.09 ± 0.011	6.16 ± 0.02

### Measurement of phosphate contents in cell-free broth using toluidine blue O dye

**Figure 2:**
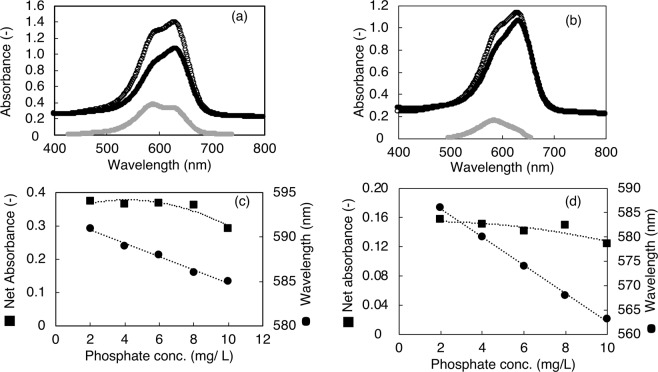
Absorbance scans of reaction product (**a** and **b**; TBO and phosphate in concentration of 2 mg/L in cell-free broth). Net absorbances and shifts in wavelengths of first peaks were used to determine calibration curves (**c**, **d**). (**a**) and (**c**) are for *L. casei* JCM 1134 while (**b**) and (**d**) are for *B. adolescentis* JCM 1275. In (**a**) and (**b**), white dots → sample, black dots → blank, and grey dots → net absorbance. In (**c**) and (**d**), black circles  → change in wavelength and black squares → net absorbance. Standard errors of observed values were found less than 2%.

We observed two peaks for the samples and their respective blanks during the absorbance scans, as shown in Fig. 2a,b, for a phosphate concentration of 2 mg/L in the cell-free broth. The wavelength of the first peak was found to be related to the phosphate concentration. The wavelength of the first peak in the net absorbance scans shifted gradually with the phosphate concentration, as shown in Fig. 2c,d. The observed trend in the absorbance as well as the wavelength shift (Fig. 2c,d) were found in agreement with the trend in the amount of phosphate accumulated as shown in Fig. 1a. The shifts in the wavelength of the first peak and the absorbance of the first peak in the net absorbance scans (with and without phosphate in the salt solution) of the reaction product owing to the reaction between the studied cell-free broths and TBO dye were used to determine the phosphate calibration curves (2 to 10 mg/L), which are shown in Fig. 2c,d. Both trends indicated a sharp shift during the first hour of incubation, followed by gradual shifts, confirming that there exists an inverse correlation: the absorbance and the shift in wavelength of the first peak with the phosphate content of the samples. Therefore, calibration curves were prepared for the K_2_HPO_4_ content in the supernatant of cell free assay salt solution. The calculations used to obtain the calibration curves that are described in Supplementary Data S1.

$x=391.61+1.0142\,\{-0.0011\,{y}^{2}-(\frac{z}{2.706})\}$ for the L11 isolate of *L. casei* JCM 1134 (R^2^ = 0.98736) and $x=104.83+0.999\{-0.0003\,{y}^{2}-(\frac{z}{1775789.9})\}$ for the B8 isolate of *B. adolescentis* JCM 1275(R^2^ = 0.99846), where *x* is the phosphate concentration (mg/L), *y* is the wavelength of the first peak, and *z* is the absorbance corresponding to the wavelength of the first peak.

### Validation

The calibration curves were validated by comparing the phosphate concentrations determined using the TBO-based phosphate estimation method with those obtained by the ascorbic acid method for cell-free samples (Tables 4 and 5). The lower detection limits of phosphate estimation for the L11 isolate of *L. casei* JCM 1134 and the B8 isolate of *B. adolescentis* JCM 1275 were 1.24 and 0.4 mg/L, respectively. Thus, the calibration curves were confirmed at a confidence level of 95% (p > 0.05).

**Table 4:** Phosphate contents (mg/mL, ascorbic acid method) were compared with TBO-based phosphate estimates at different time intervals of incubation in assay salt solution for L11 isolate of *L. casei* JCM 1134 where p < 0.05, t critical value = 18.512 with double-tailed test, F-critical = 12.706, and n = 3.

Time (h)	TBO P estimation	Ascorbic acid method	F value	t-value	P-value
1	9.73 ± 0.02	9.52 ± 0.03	6.90	0.174	0.120
2	5.73 ± 0.03	5.50 ± 0.03	8.80	0.266	0.097
4	0.40 ± 0.02	0.38 ± 0.03	9.85	0.156	0.088
6	−2.27 ± 0.03	−2.17 ± 0.01	4.11	0.250	0.180

**Table 5:** Phosphate contents (mg/mL, ascorbic acid method) were compared with TBO-based phosphate estimates at different time intervals of incubation in assay salt solution for B8 isolate of *B. adolescentis* JCM 1275 where p < 0.05, t critical value = 12.706, F-critical = 18.512 with double-tailed test, and n = 3.

Time (h)	TBO P estimation	Ascorbic acid method	F value	t-value	P-value
1	6.75 ± 0.01	6.65 ± 0.03	0.89	0.384	0.445
2	4.68 ± 0.01	4.50 ± 0.02	4.39	0.217	0.171
4	1.24 ± 0.02	1.18 ± 0.02	1.02	0.602	0.418

## Discussion

To prepare the phosphate-deficient cells, 8 h of incubation was found to be sufficient. The obtained data (Table 2) showed that the phosphate content reduced by almost 50% for both intestinal cells. There was no further utilisation of the stored phosphate was observed, probably because the remaining phosphate were present in an inert form. Moreover, significant cell loss occurred after 8 h of incubation.

It is critical to monitor sugar maintenance in phosphate-deficient and phosphate-containing cells because subsequent incubation in a salt solution involves phosphate accumulation (phosphate-deficient cells) or the utilisation of the stored phosphate (phosphate-containing cells); these phenomena in conjunction with sugar metabolism are responsible for maintaining cell viability. Therefore, the sugar contents of the phosphate-deficient and phosphate-containing cells must remain unchanged before incubation in the salt solution.

In the case of the L11 isolate of *L. casei* JCM 1134, the OD remained unchanged for up to 6 h of incubation both with and without the phosphate salt solution (Table 3). After 8 h of incubation, the OD subsequently decreased in both cases, while the pH increased sharply, indicating a sudden release of basic ions, possibly phosphate ions, into the phosphate-deficient broth. This was confirmed by the phosphate content as observed at 8 h of incubation in the phosphate-deficient broth (Fig. 1a). Therefore, 6 h of incubation was considered appropriate for further experiments. However, at 8 h of incubation, the decrease in the OD did not significantly alter the pH value of the phosphate-containing broth (Table 3), indicating a reduction in the amount of phosphate stored. A similar trend was observed for the B8 isolate of *B. adolescentis* JCM 1275, as shown in Fig. 1a and Table 3. For the B8 isolate of *B. adolescentis* JCM 1275, a suitable incubation time for estimating the phosphate-accumulation capacity and avoiding significant cell loss was found to be 4 h (Fig. 1a), as the release of phosphate was observed after 6 h of incubation. In comparison, the L11 isolate of *L. casei* JCM 1134 acclimatised better to the altered environment and accumulated more phosphate from the designed system than did the B8 isolate of *B. adolescentis* JCM 1275. The initial accumulation (after 1 h of incubation) of phosphate was rapid, in contrast to the subsequent trend of the phosphate content increasing gradually (Fig. 1a). The effect of the altered conditions corresponding to the designed system could explain the observed differences in the acclimatisation and phosphate-accumulation capabilities of the studied cells.

In the net absorbance scans, two peaks were observed, which could be attributed to the formation of different TBO and phosphate complexes in the autoclave (conditions: 121 °C for 15 min). Moreover, neither the shift in the second peak nor the corresponding absorbance was related to the observed trend in the phosphate content. In contrast, the absorbance corresponding to the first peak and the associated wavelength change were incorporated into the phosphate estimation model since these parameters exhibited an association with the phosphate content.

Previous studies on phosphate removal have analysed waste water samples in detail. Increases in environmental phosphate levels can be attributed to the high phosphate loads in wastewater sludge systems. High-efficiency biological phosphate removal systems as well as chemical precipitation methods have been designed to solve the global issue of increased phosphate deposition in wastewater. Several parameters affect the phosphate-accumulation capabilities of PAOs, such as any sudden environmental shifts or changes in the nutrient composition, and the anaerobic/aerobic cycle time, as well as the pH, temperature, phosphate content, sulfate level, ammonium ion concentration, macronutrient content, and essential trace metal (K^+^, Na^+^, Mg^+2^, Ca^+2^) concentrations^10–13^.

The poly-P content can be determined by ion chromatography^8^ and inductively coupled plasma atomic emission spectrometry^13^. However, these techniques require a preliminary digestion step^14^ and are also costly. Researchers have attempted to extract poly-P by digesting cells with chloroform, phenol, and amyl alcohol. TBO dye is then reacted with the extracted poly-P, and the absorbance is measured^15^. The challenges in this-cited studies (discussed in introduction section) need to be addressed, additionally, no trend was observed at any wavelength in the absorbance scan of the reaction product (for the TBO and phosphate-containing samples). On the other hand, in the present study, we observed shifts in the first peak of the absorbance scan, with the shifts being inversely proportional to the phosphate concentration (K_2_HPO_4_, which is more suitable for use as a standard than any specific polymer of P) in the sample. In addition, we could enhance the sensitivity of the method by increasing the reaction temperature (see Supplementary Fig. S2). Further, because we used cell-free microbial metabolites as the blanks for each cultivation time step, the effects of any interfering agents as well as the false positive or negative effects arising from metabolites could be prevented.

A comparison of the results obtained using the proposed TBO-based phosphate estimation method and those obtained by the ascorbic acid method confirmed the advantages of the former. The ascorbic acid method involves the use of harsh chemicals, making it hard to dispose the reaction products, while in the case of the proposed method, the only material that needs to be disposed is the TBO dye, which is safe and can be handled using a general waste disposal system. Further, the required concentration of TBO is very low, in contrast to the high concentrations of the chemicals used in the ascorbic acid method. Moreover, in the TBO-based method, cell lysis and extraction are avoided, and the supernatant of the broth can be used for further studies. The proposed phosphate estimation method is highly reliable because of the use of almost perfect blanks in terms of the reactivity of any interfering agents and the relative concentrations of the metabolites. Furthermore, comparisons with other TBO-based methods^16^ for phosphate estimation indicated that the proposed method has better reliability and specificity. In this study, TBO was reacted with the supernatant of the broth in an autoclave (at 121 °C for 15 min); this significantly increased the sensitivity of the method with respect to that for reactions occurring at room temperature.

The proposed phosphate estimation method is simple, reliable, specific, and affordable and does not require the use of harsh chemicals. Further, this novel strategy of estimating the phosphate content using supernatants instead of extraction procedures solves the problems of cell lysis and undesired modifications. To determine the maximum phosphate-accumulation capacities of potential PAOs  using the designed system, phosphate-deficient cells were constructed, and their phosphate-accumulation capacities were measured in a specifically designed salt solution. This helped to prevent the issues arising from interfering agents, as perfect blanks were used.

The proposed TBO-based phosphate method can be employed to evaluate the inherited capacities of PAOs to accumulate phosphate and thus assess their suitability for use in the prevention of hyperphosphatemia. However, the method must be standardized so that it meets the other PAO requirements. This would further increase the applicability of the method. In future studies, we intend to explore phosphate removal from phosphate-rich foods or complex food systems using PAOs.

## Methods

### Chemicals and equipment used

MRS (De Man, Rogosa and Sharpe, Becton Dickinson Difco, Japan), hipolypeptone (Nihon Seiyaku, Japan), beef extract (MP Biomedicals, LLC, France), yeast extract (Becton Dickinson Difco, Japan), and TBO (Waldeck, Münster) were used to prepare the growth medium. Glucose, Tween 80, K_2_HPO_4_, sodium ascorbate, L-cysteine. HCl, NaNO_3_, MgSO_4_, KH_2_PO_4_, NaH_2_PO_4_, NaCl, citric acid, sodium citrate, and 4ʹ,6-diamidino-2-phenylindole (DAPI) were used in the experiments and were procured from Fujifilm Wako Pure Chemical Co., Japan. A spectrophotometer (Shimadzu, Japan), fluorescence microscope DMRXA/RD (Leica Microsystems, Japan), and centrifuge system (Kokusan, Japan) were also used in the experiments. The water used in all the experiments was deionised and double distilled prior to use.

### Sub-culturing and maintenance of probiotics

The L11 isolate of *L. casei* JCM 1134 and the B8 isolate of *B. adolescentis* JCM 1275 were used throughout this study. Starter cultures were inoculated from frozen glycerol stock cultures into an Erlenmeyer flask (200 mL) containing 100 mL of sterilised MRS medium and bifidobacterium medium (w/v: hipolypeptone 1%, beef extract 0.5%, yeast extract 0.5%, glucose 1%, Tween 80 0.1%, K_2_HPO_4_ 0.3%, filter-sterilised sodium ascorbate 1%, and L-cysteine HCl 0.05%), grown for 24 h at 37 °C, and subcultured for further studies.

### Transformation media used

Transformation media were used to transform the phosphate-containing probiotics into phosphate-deficient ones. The constituents of the transformation media are listed in Table 6, and the media were prepared using a 0.1 M citrate buffer. Half the volume of the active L11 isolate of *L. casei* JCM 1134 and the B8 isolate of *B. adolescentis* JCM 1275 were transferred to the filter-sterilised transformation media while keeping the OD the same as that of the previous culture broth, and samples were collected after 0, 1, 2, 4, 6, and 8 h of incubation at 37 °C. The remaining untreated cells were incubated in an ice box, and samples were collected after 0 and 8 h of incubation. The OD of the collected samples was analysed at 680 nm while using DAPI staining, the cell viability was monitored as well as the content of the stored phosphate. The starting and final samples incubated in both the ice box and the transformation media were analysed in terms of their CFU/L values, total sugar and phosphate contents using the spread plate method, phenol–sulfuric acid and ascorbic acid methods respectively.

**Table 6:** Composition of transformation media in 0.1M citrate buffer (pH 6.0).

Ingredients % (w/v)	L11 of *L. casei* JCM 1134	B8 of *B. adolescentis* JCM 1275
Glucose	5.0	3.0
NaNO_3_	0.5	0.25
MgSO_4_	0.05	0.05
Tween 80	0.1	0.1
pH	6.0	6.0

### Salt solutions used

Two types of salt solutions were prepared. The media with and without phosphate served as the test and blank samples, respectively. However, the phosphate-deficient salt solution was supplemented with NaCl to maintain the osmolarity of the broth. The composition of the salt solution was as per previous studies^10–12^, and it was prepared in a 0.1 M citrate buffer, as shown in Table 7. The cells incubated in the transformation media were transferred to filter-sterilised phosphate-containing salt solutions (test samples), while the cells incubated in the ice box were transferred to a filter-sterilised salt solution (phosphate-deficient) containing NaCl (blank samples) while ensuring that the OD was the same as that of the previous culture broth. Samples were collected after 0, 1, 2, 4, 6, and 8 h of incubation at 37 °C. The collected samples were analysed by measuring the OD at 680 nm and the phosphate content of the supernatants (the samples were centrifuged at 1,915 × *g*  for 5 min) in order to monitor cell viability and the remaining phosphate content, respectively. The starting and final samples were analysed using DAPI staining.

**Table 7:** Composition of assay salt solution in 0.1M citrate buffer (pH 6.0).

Ingredients % (w/v)	L11 of *L. casei* JCM 1134	B8 of *B. adolescentis* JCM 1275
NaNO_3_	0.8	0.8
MgSO_4_	0.6	0.6
K_2_HPO_4_/NaCl	2.0/1.0	1.3/0.6
Tween 80	0.1	0.1
pH	6.0	6.0

### Measurement of phosphate content using TBO-based estimation method

The samples collected from the salt solutions were centrifuged at 1,915 × *g* for 5 min, and the supernatants (1 mL) were reacted with 0.00125% (w/v) TBO in an autoclave (at 121°C for 15 min). The samples were then cooled and centrifuged at 5,320 × *g* for 10 min, and the supernatants were subjected to an absorbance scan from 400 to 800 nm. The absorbance scans of the test samples were subtracted from those of the blank samples, the trend related to phosphate concentration in the first peaks of absorbance scans were determined and subsequently phosphate contents were estimated using calibration curve, and a graph of the phosphate content as a function of time was plotted (Fig. 1a).

### Calibration curve for TBO-based phosphate estimation method

To determine the phosphate content in the supernatant of the culture, a phosphate calibration curve was drawn. The phosphate content in the supernatant of the last samples were estimated using the ascorbic acid method. The concentration of K_2_HPO_4_ in the supernatant of the last sample was adjusted to different levels (2, 4, 6, 8, and 10 mg/mL), and the supernatant was reacted with TBO (0.00125% w/v) in an autoclave (at 121 °C for 15 min). After the samples had been allowed to cool to room temperature and then subjected to centrifugation at 5,320 × *g* for 10 min, their absorbances were recorded from 400 to 800 nm. The absorbance scans of the test samples were subtracted from those of the blank ones, and the absorbances and wavelengths of the first peaks were determined. Tables 4 and 5 shows the phosphate concentrations for the L11 isolate of *L. casei* JCM 1134 and the B8 isolate of *B. adolescentis* JCM 1275.

### Measurement of growth characteristics

The samples were suitably diluted, and their absorbances (as OD) were determined at 680 nm using an ultraviolet-visible spectrophotometer. The CFU/L value was estimated by aseptically spread-plating the diluted samples on an MRS agar medium. The sugar content was estimated using the phenol–sulfuric acid method^17^.

### Measurement of phosphate concentrations using DAPI and ascorbic acid methods

Broth samples (245 μL) were mixed with 12.5 μl of DAPI stain (1000 μg/mL of 0.025 M Tris-HCl buffer). Next, glass slides of the samples were mounted and analyzed under an ultraviolet-fluorescence microscope DMRXA/RD (Leica Microsystems, Japan. Software: LAS X) with high sensitivity cooled CCD color camera VB-700 (Keyence, Japan) and fluorescence digital microscope camera controller VB-7000 (Keyence). The fluorescent images for microbes were 300 dpi and captured at room temperature in their respective salt solutions for the absorption wavelength of 385 nm and emission wavelength of 461 nm (DAPI dye). The ascorbic acid method was used to estimate the phosphate concentrations in the collected samples^18^.

### Statistical analysis

All the experiments were performed in triplicate (n = 3). An analysis of variance (ANOVA) was performed using MS Excel 2016 (version 15.26) to determine the significant differences (p < 0.05). Error bars are included for all the numerical data.

## Supplementary information

Dataset 1 S1, S2

## Data Availability

All data generated or analysed during this study are included in this article.
