# Supplementary material for: Estimation of microbial phosphate-accumulation abilities
Source: Sci Rep. 2019 Mar 19;9:4879. doi: 10.1038/s41598-018-37752-8 (PMC6425018; doi:10.1038/s41598-018-37752-8)
Supplement: Supplementary file 1 — Dataset 1 S1, S2 [file 41598_2018_37752_MOESM1_ESM.pdf]

Estimation of microbial phosphate-accumulation abilities

Ajeeta Anand[1], Hideki Aoyagi[1, 2*]
[1]Institute of Life Sciences and Bioengineering,
Graduate School of Life and Environmental Sciences,
University of Tsukuba, Tsukuba, Ibaraki 305-8572, Japan

[2]Faculty of Life and Environmental Sciences,
University of Tsukuba, Tsukuba, Ibaraki 305-8572, Japan

*Corresponding author
Email: aoyagi.hideki.ge@u.tsukuba.ac.jp
Tel: +81-298537212, Fax: +81-298534605

S1. Calculation of Phosphate using TBO method

- Calibration curve was drawn using K_2HPO_4 conc. (2, 4, 6, 8, 10 mg/ L) as phosphate source and the absorbance scan of reaction product of Toluidine blue O and phosphate was analyzed in terms of absorbance and wavelength of first peak.

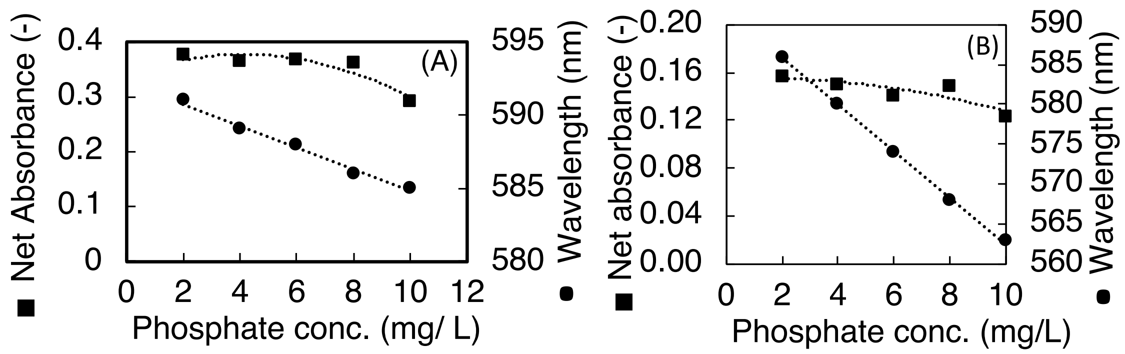

S1.1 Change in absorbance and wavelength with phosphate concentration. (A)→ *L. casei* and (B)→ *B. adolescentis*

- We observed that the phosphate conc. depend on net absorbance and wavelength of peak detected.

For *Lactobacillus casei* JCM 1134 (L11), 2 equations were made:

For Absorbance, z= $0.0023x^2+0.019x+0.3387$, $R^2=0.8666$, Quadratic equation

For wavelength, y=$-0.75x+592.3$, $R^2=0.9868$, linear

Where, x= Phosphate conc. (mg/ L), y= Wavelength of 1st peak (nm) and z= Absorbance (-)

Quadratic and linear equations were combined using replacement method to know x, phosphate conc.

> ➢ **Replacement method:** P estimation in supernatant of assay solution for L11
>
> $$\boldsymbol{x} = 391.61 + 1.0142\{ -0.0011\boldsymbol{y}^2 - \left(\frac{\boldsymbol{z}}{2.706}\right)\}$$ $R^2 = 0.98736$
>
> (predictable)

Similarly, for *Bifidobacterium adolescentis* JCM 1275 (B8),

➢ **Similarly for *Bifidobacterium adolescentis*,** P estimation in supernatant of assay solution:

$$\boldsymbol{x} = 104.83 + 0.999\{-0.0003\boldsymbol{y}^2 - (\frac{\boldsymbol{z}}{1775789.9})\}$$

$R^2 = 0.99846$

S2. Supplementary Figure

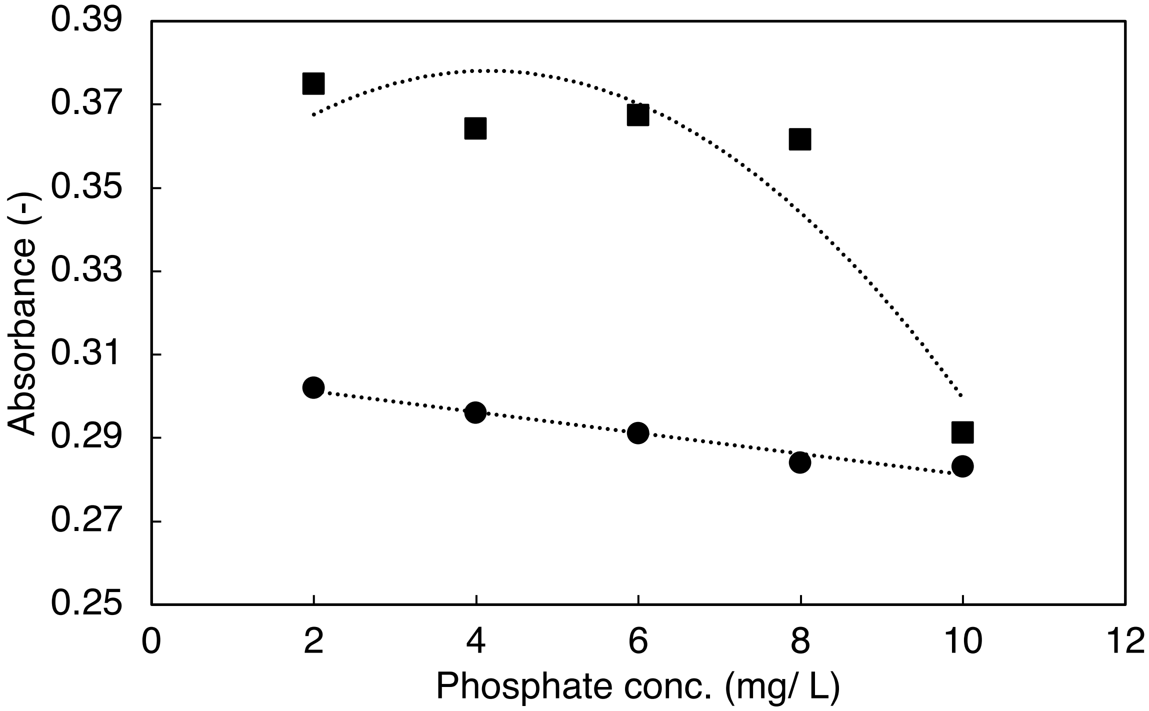

S2. Effect of elevated temperature (autoclave) on absorbance sensitivity of reaction product in scan (400-700 nm).

Black circle: with autoclave, black square: no autoclave
